# Next‐generation signaling: Powering the future of publications in the *Journal of Cell Communication and Signaling*

**DOI:** 10.1002/ccs3.70069

**Published:** 2026-03-06

**Authors:** Brahim Chaqour

**Affiliations:** ^1^ Department of Molecular Ophthalmology Perelman School of Medicine University of Pennsylvania Philadelphia Pennsylvania USA

**Keywords:** cell communication, cell signaling, editorial, *JCCS*, journal growth, Research Exchange

## Abstract

The *Journal of Cell Communication and Signaling* (*JCCS*) is dedicated to advancing understanding of how intracellular and extracellular signaling coordinate environmental sensing, gene regulation, and tissue homeostasis and how their dysregulation or misinterpretation contributes to disease. Reflecting on my first year as editor‐in‐chief, I am pleased to highlight the journal's continued progress and strategic development. Submissions, readership, and article diversity have grown steadily, driven by the commitment of authors, reviewers, editors, and publishing staff. *JCCS* remains selective yet inclusive in scope, emphasizing high‐quality studies that illuminate signaling processes in health and disease. Ongoing initiatives to reduce decision times and enhance the expertise and global representation of our editorial and reviewer communities reinforce peer review as a rigorous collaborative enterprise. A key milestone ahead is the transition of *JCCS* manuscript handling to Wiley's Research Exchange (REX) platform, which integrates submission, integrity screening, and peer review within a unified system. This platform leverages artificial intelligence tools, metadata extraction, and ORCID integration to improve efficiency, transparency, and ethical oversight. Looking forward, *JCCS* aims to strengthen peer review, introduce special issues on emerging topics in cell communication, and foster greater international engagement to advance the field's scientific and collaborative frontiers.

## 1 EDITORIAL

My first‐year reflection as editor‐in‐chief of *JCCS* is a heartfelt word of thanks to the many who worked, contributed, and supported *JCCS* this my first year as EiC. Over the past year, *JCCS* depended on the trust of authors who chose *JCCS* to publish their work, the thoughtful reviews by busy colleagues who volunteered their time to contribute to the review process, the steady support of the editorial board who were responsive to my requests and assignments, and the staff members at Wiley who worked tirelessly to maintain the journal's standing in the publishing landscape. This collective effort has allowed *JCCS* to maintain high standard while remaining open to emerging ideas and scientific discoveries.

Over the past 12 months, *JCCS* oversaw a substantial increase of submissions and published a diverse set of articles that highlighted new research findings on intracellular and extracellular signaling networks and their impact in disease pathogenesis and treatments. From the outset, the editorial decisions have been guided by the goal of publishing rigorous relevant research that advances both scientific understanding and practical impact on human health. In this rapidly evolving landscape, the journal has sought to balance continuity with innovation preserving its core identity on research in cell communication and signaling while welcoming new topics using modern technologies. It is refreshing to see that the journal continued its course of sustained growth and competitiveness as seen by increased number of submissions, publications views, and downloads while maintaining a competitive acceptance rate of 17%. I am excited to pursue the work that we started this year and expand the journal's readership, continue to attract high quality scientific contributions while ensuring transparency, and rigor in scientific reporting and a hassle free‐manuscript processing and decision‐making processes.

I started my EiC tenure working closely with the editorial board member and the executive editors of the journal to tackle the backlog of manuscripts in the peer review system speeding up the review process and alleviating the workload of our editors while preventing unnecessary delays for the streams of submitted manuscripts. Our partly updated editorial team has and continues to play a crucial role in meeting this ambitious goal. I am aware of the challenge that editors of all journals face to find suitable reviewers for papers advanced for review and publications in the journal. However, all our editors have lived up to this challenge, and the journal could not be as successful if not for the wide breath of expertise of its new and experienced editorial board members and dedications of its geographically diverse reviewers who volunteer their service to support the scholarly mission of *JCCS*. Researchers have every incentive to submit manuscripts but little or no incentive to review those submitted by others. However, because most reviewers are also authors, they can evidently benefit from critical appraisal of their work by others as much as they do when they receive constructive feedback on their own manuscripts. This reciprocal dynamic underscores the collaborative foundation of research communications. The peer review process functions most effectively when researchers recognize their dual roles as both contributors and evaluators within the scientific community. By participating conscientiously as reviewers, researchers uphold the standards of their field, foster intellectual rigor, and strengthen the integrity of the publication ecosystem that, in turn, ensures fair and informed evaluation of their own work. Continued efforts will be pursued in the upcoming year to expand *JCCS* reviewers' pool and the global representation and expertise of the editorial board.

*JCCS* editors assume primary responsibility for determining the final disposition of a manuscript. I am honored to work with editors who take their duties seriously and always make sure to integrate disparate perspectives from reviewers, exercise informed judgment, and provide authors with an integrated and balanced directive that advances the integrity and clarity of the publication process. I am committed to continue working with all *JCCS* associate and executive editors to enforce this effective editorial practice which greatly assists the authors in strengthening their paper for resubmission to the Journal. Along these lines and to sustain and enhance this high standard of editorial excellence, *JCCS* is committed to continuously expanding its pool of highly effective and talented editors. By engaging a diverse group of scientists with recognized expertise, sound judgment, and a dedication to ethical editorial practice, the Journal ensures that each submission benefits from thoughtful, fair, and authoritative evaluation. *JCCS* has open invitations to scientists at all career levels invested in diverse aspects of cell communication and signaling to submit their request to contribute to the editorial and/or review process of the journal by emailing *JCCS* EiC (JCCS-eic@outlook.com). This deliberate investment in editorial capacity not only supports the integrity of the review process but also fosters innovation and inclusivity within the journal's readership and the scientific community at large.

As we wrap up 2025 and to better support authors and editors' needs, *JCCS* will transition from Editorial Manager to Research Exchange. Research Exchange (also abbreviated as REX) is Wiley's new online platform designed to manage the submission, screening, and peer review of manuscripts serving as a modern alternative to legacy systems, such as Editorial Manager and ScholarOne previously used by many publishers.

REX integrates the three core components of manuscript submission, integrity screening, and peer review into a single workflow, reducing the need for multiple and somewhat disconnected processes. *JCCS* is up for migration of manuscript handling into REX this upcoming year 2026.

REX has several key capabilities, including (1) providing a unified submission interface with automated metadata extraction, and minimum manual data entry for authors, making manuscript submission user friendly, and as easy and speedy as possible; (2) use of artificial intelligence (AI)‐powered tools and rules‐dependent screening of submitted manuscripts to detect potential compliance and ethics and integrity issues before peer review with 30 automated integrity checks, including Wiley's proprietary Papermill Detection Tool, to flag potential integrity challenges; and (3) use of REX review that support different editorial workflows, reviewer selection tools and cross journal transfer. REX is designed to support identification of suitable reviewers using AI to suggest experts based on manuscript keywords and bibliography and publication history from internal and external databases for quality peer review. In addition, REX connects with ORCID for validated identities and affiliation and checking for conflict of interest. Altogether, REX screening system is designed to support editorial decisions enabling editors to make more informed decisions. By adopting REX platform, *JCCS* is embracing a more integrated, efficient, and transparent publishing workflow that enhances the experience for authors, reviewers, and editors alike. This transition marks an important step toward streamlining editorial processes and strengthening the journal's commitment to research integrity and innovation in research publishing.

Looking ahead, priorities for the coming year will also include further strengthening the review process, exploring and developing new thematic issues that address emerging challenges and opportunities in research of cell–cell and cell‐matrix communication and encouraging readers and researchers to submit their best work to *JCCS*, volunteer as reviewers, propose ideas, and topics for special issues of *JCCS* and share feedback on how the journal can continue to excel in evaluating and publishing outstanding research publications to better human health.

## CONFLICT OF INTEREST STATEMENT

Dr. Brahim Chaqour is the editor‐in‐chief of *JCCS*.

## ETHICAL STATEMENT

Not applicable.

## CONSENT FOR PUBLICATION

Not applicable.

## Data Availability

Data sharing is not applicable to this article as no new data were created or analyzed in this study.

